# Early neurodevelopmental brain perfusion abnormalities and functional connectivity findings in infants with Prader-Willi syndrome

**DOI:** 10.1186/s11689-026-09690-4

**Published:** 2026-04-06

**Authors:** Jennifer Boisgontier, Sarah Charpy, Graziella Pinto, Volodia Dangouloff-Ros, Ludovic Fillon, Ana Saitovitch, Sara Cabet, Khawla Aljabali, Aurélie Fabre, Gwenaëlle Diene, Marion Valette, David Cohen, Monica Zilbovicius, Maïthé Tauber, Nathalie Boddaert

**Affiliations:** 1 Department of Pediatric Radiology, Necker-Enfants Malades Hospital, AP- HP, Paris, F-75015 France; 2 Paris Cité University, Imagine Institute, INSERM U1163, Paris, France; 3 Department of Endocrinology, Diabetology and Gynecology, Necker- Enfants Malades Hospital, AP-HP, Paris, France; 4 Regional Mobile Genetics Consultation Unit, Elan Retrouvé Foundation, Paris, France; 5 National Reference Center for Rare Diseases PRADORT (Prader-Willi syndrome and Other Rare Obesities with Eating Behavior Disorders), University of Toulouse III - Paul Sabatier, Toulouse, France; 6 Center for Epidemiology and Population Health Research (CERPOP), INSERM UMR1295, University of Toulouse III - Paul Sabatier, Toulouse, France; 7 Department of Child and Adolescent Psychiatry, IDEAL Institute, AP-HP, Sorbonne University, Paris, France; 8 CNRS UMR 7222, Institute of Intelligent Systems and Robotics (ISIR), Sorbonne University, Paris, France; 9 École Normale Supérieure Paris-Saclay, Inserm U1299, ERL “Developmental Trajectories and Psychiatry,” University of Paris-Saclay, University of Paris, CNRS, Borelli Center, Gif-sur-Yvette, France; 10 Institute for Infectious and Inflammatory Diseases (Infinity), INSERM UMR1291, CNRS UMR5051, University of Toulouse III - Paul Sabatier, Toulouse, France

**Keywords:** Prader-Willi syndrome, Infancy, Neurodevelopment, Arterial spin labeling, Cerebral blood flow, Resting-state functional MRI, Functional connectivity

## Abstract

**Background:**

Prader-Willi syndrome (PWS) is a neurodevelopmental disorder characterized by distinct eating behaviors that evolve from early feeding difficulties to later hyperphagia. Early brain abnormalities remain poorly understood, with no data on brain perfusion during infancy. This study investigated early brain perfusion and functional connectivity in infants with PWS and their associations with feeding and social functioning.

**Method:**

Twenty-seven infants (mean age 3 months) were included in this prospective study. Thirteen genetically confirmed PWS infants (mean age 2.7 ± 1.2 months) underwent 3T multimodal MRI combined with structural imaging, arterial spin labeling (ASL) to quantify cerebral blood flow (CBF) at rest, and resting-state functional MRI to assess functional connectivity. Oral-motor and social functioning were evaluated via the Neonatal Oral-Motor Assessment Scale (NOMAS) and Coding Interactive Behavior (CIB) scale. Group differences in CBF between PWS infants and 14 age-matched controls (mean age 3.5 ± 1.1 months) were investigated via whole-brain voxelwise analysis and linear regression, controlling for age and sex (*p* ≤ 0.05, FWE-corrected). Within the PWS group, associations between imaging and clinical data were analyzed via age-adjusted linear regressions, corrected for multiple comparisons using the false discovery rate procedure.

**Results:**

Compared with controls, infants with PWS presented significantly increased CBF in the insula-superior temporal region, striatum-pallidum, and anterior cingulate cortex (p_(FWE)_ ≤ 0.05). Within the PWS group, the anterior cingulate CBF was significantly associated with oral-motor performance (p_(FDR)_ = 0.04) and birth weight (p_(FDR)_ = 0.03). The functional connectivity between the insula-temporal region and the striatum pallidum was significantly associated with unacylated ghrelin levels (p_(FDR)_ = 0.04), whereas the interhemispheric striatum-pallidum functional connectivity was significantly associated with social functioning (p_(FDR)_ = 0.01).

**Conclusions:**

This multimodal MRI study identified early brain hyperperfusion in infants with PWS, preceding the onset of hallmark symptoms. Perfusion in the anterior cingulate cortex is related to early feeding and growth, and functional connectivity within the insular and striatal regions is linked to social and metabolic measures, suggesting that these associations develop early in life. These findings provide a basis for future longitudinal studies to clarify how early patterns of brain function are related to developmental outcomes.

**Supplementary Information:**

The online version contains supplementary material available at 10.1186/s11689-026-09690-4.

## Background

Prader-Willi syndrome (PWS) (OMIM 176270) is a genetic neurodevelopmental disorder caused by the lack of expression of paternally inherited imprinted genes on chromosome 15q11-q13, with a prevalence of approximately 1 in 20,000 live births [1]. The syndrome is characterized by evolving patterns of eating behavior across developmental stages [2]. In the neonatal period, severe hypotonia and weak sucking often lead to feeding difficulties requiring nasogastric support [3]. By two years of age, children typically develop a pathologically increased appetite and impaired satiety, leading to hyperphagia and obesity. Cognitive and behavioral difficulties, including learning and speech delays, poor frustration tolerance, temper outbursts, obsessive-compulsive symptoms, and anxiety, are also part of the PWS phenotype [4]. Approximately 30% of patients present features of autism spectrum disorder, including social and communication difficulties and repetitive behaviors [5]. Endocrine abnormalities such as growth hormone deficiency and hypogonadism reflect hypothalamic dysfunction, a core feature of the syndrome [6, 7].

Neuroimaging studies in children, adolescents, and adults with PWS have revealed abnormalities in several brain regions, particularly those involved in hunger and appetite regulation, including the hypothalamus (satiety and energy balance), prefrontal cortex (decision-making), striatum and amygdala (reward processing), and insula (interoception and hunger) [8–10]. However, little is known about early brain development, especially in infants with PWS younger than six months. To date, only two studies have examined brain changes in infants with PWS. First, functional magnetic resonance imaging (MRI) revealed that intranasal oxytocin enhanced frontal connectivity and improved feeding and social interaction [11]. The second, on the basis of structural MRI, examined genotype-phenotype associations in a cohort of newborns with PWS and reported reduced frontal gray matter volume [12]. Together, these findings suggest early brain alterations in PWS but underscore the need for broader multimodal approaches. To our knowledge, no study has examined brain perfusion during infancy, limiting the current understanding of early brain function in PWS.

Multimodal brain imaging offers valuable opportunities to advance the understanding of early neurodevelopment. Among the available approaches, noninvasive MRI techniques such as arterial spin labeling (ASL) and resting-state functional MRI are particularly suitable for use in infants, whether healthy or affected by neurodevelopmental conditions [13, 14]. ASL has been successfully applied to detect functional perfusion differences in autism spectrum disorder, reinforcing its relevance for investigating early brain alterations in PWS [15]. ASL quantifies cerebral blood flow (CBF) at rest, providing a direct measure of brain perfusion, whereas resting-state functional MRI evaluates functional connectivity, defined as the temporal correlation of spontaneous brain activity between distinct regions during rest [16, 17]. Together, these methods offer a powerful framework to characterize developmental brain function in early infancy and are particularly well suited to explore the earliest stages of pathophysiology. Such neuroimaging approaches are increasingly recognized as valuable tools for quantifying early functional alterations and developing objective outcome measures in individuals with neurodevelopmental disorders [18].

This study aimed to characterize early functional brain organization in infants with PWS younger than six months via a multimodal approach combining ASL and resting-state functional MRI. We compared CBF at rest between infants with PWS and age-matched controls. In the PWS group, we investigated functional connectivity patterns and examined associations between both imaging metrics (CBF at rest and functional connectivity) and clinical measures of feeding and social functioning.

## Methods

### Study design

This prospective observational neuroimaging study included infants with PWS who were enrolled through a multicenter therapeutic trial including brain MRI (NCT04283578). The present analysis focused on data from one participating center, Necker-Enfants Malades Hospital (AP-HP, Paris, France), where recruitment took place between 2020 and 2021. Importantly, no therapeutic intervention was administered to the infants with PWS included in this analysis; only clinical and neuroimaging data were collected and analyzed. All MRI scans were acquired during natural sleep without sedation, using a standardized clinical natural-sleep MRI protocol routinely applied. The sample size was determined by feasibility and clinical data availability, in line with recommendations for early-phase neurodevelopmental imaging studies [19].

### Study population

Fifteen infants with genetically confirmed PWS (8 males; mean age = 2.7 ± 1.2 months; range: 1 to 5.5 months) were prospectively enrolled. The inclusion criteria were as follows: (1) genetically confirmed PWS; (2) age younger than six months at MRI; and (3) the hallmark neonatal presentation of PWS, defined by severe hypotonia, weak sucking, and feeding difficulties. None of the infants had received therapeutic intervention before imaging.

Sixteen control infants (8 males; mean age = 3.4 ± 1.4 months; range: 1.5 to 5.5 months) were retrospectively selected from the institutional imaging database at approximately a 1:1 ratio with infants with PWS, matched for age. These infants had undergone MRI including ASL sequence as part of routine clinical care for non-neurological indications. The exclusion criteria were prematurity, congenital anomalies, neurological disorders, abnormal brain MRI, and abnormal psychomotor development. The absence of abnormalities was confirmed by two experienced pediatric neuroradiologists (N.B., V.D.R.), and typical neurodevelopment was verified through follow-up consultations. Individual demographics and clinical characteristics are summarized in Table S2 for the control group.

### Ethical approval and consent to participate

The inclusion of infants with PWS was approved by the institutional Ethics Committee (EUDRACT: 2019-6002385-12) and conducted in accordance with the Declaration of Helsinki. Written informed consent for participation and MRI acquisition was obtained from both parents of all the participating infants.

Institutional Review Board approval was not required for the inclusion of control infants, as fully anonymized MRI data obtained during routine clinical care were retrospectively used. Parents were informed that these data could be used for research purposes and had the opportunity to decline their use.

### Clinical assessments

Clinical assessments were performed by the same expert pediatric endocrinologist (G.P.) to provide standardized measures of early feeding and social functioning, ensuring consistency across participants and enabling correlation with imaging data. Assessments were scheduled close to the MRI session to ensure temporal alignment between the clinical and imaging measures. All the clinical tools used have been previously published and validated for use in infants and are developmentally appropriate and clinically relevant for assessing early features of PWS [11, 20]. Briefly, feeding ability was assessed with the Neonatal Oral-Motor Assessment Scale (NOMAS), which rates jaw and tongue movements during nutritive sucking and provides an index of oral-motor performance [21]. Social functioning was assessed via the Coding Interactive Behavior (CIB) scale, an observational measure of parent-infant interaction comprising three domains and six subscales: child (Child State, Social Engagement, Negative Emotionality), parent (Sensitivity, Intrusiveness), and dyad (Reciprocity, Negative States) [22, 23]. In addition, a videofluoroscopic swallowing study (VFSS) was performed in infants with clinical signs of swallowing dysfunction, in addition to the oral-motor evaluation [24].

### MRI data acquisition

Brain MRI was performed at Necker-Enfants Malades Hospital on a 3T scanner (Discovery MR750, GE Healthcare, Chicago, IL, USA) during natural sleep without sedation, with infants swaddled to ensure optimal acquisition. The protocol lasted approximately 20 min: all infants underwent 3D T1-weighted imaging (TR/TE = 6.9/3 ms, voxel size = 1 × 1 × 1 mm, 156 slices, acquisition time = 6 min) and 3D pseudocontinuous ASL (TR/TE = 4453/10.96 ms, voxel size = 1.875 × 1.875 × 4 mm, 40 slices, postlabeling delay = 1025 ms; labeling duration = 2500 ms, acquisition time = 4 min). Resting-state functional MRI (TR/TE = 2500/31 ms, voxel size = 3.125 × 3.125 × 3 mm, 45 slices, acquisition time = 10 min) was acquired only in the PWS group, as it was not part of the clinical protocol for controls.

### MRI data quality control

The MRI data were independently reviewed by two experienced pediatric neuroradiologists (N.B., V.D.R.) to exclude major artifacts and poor-quality scans. 3D T1, ASL, and resting-state functional MRI data underwent systematic quality control at both the raw and preprocessed stages. Coregistration of ASL and resting-state functional MRI data to native T1-weighted images was visually inspected for each subject by a pediatric neuroradiologist (V.D.R.) and an imaging engineer (L.F.) to ensure accurate anatomical alignment across both groups (see Figure S2). Spatial normalization to the age-matched 3-month UNC-BCP infant template was also visually inspected for each subject. For resting-state functional MRI, data quality was assessed using automated motion metrics in addition to visual inspection. In-scan head motion was quantified using framewise displacement (FD), derived from the six rigid-body motion parameters estimated during motion correction with Motion Correction using FMRIB’s Linear Image Registration Tool (MCFLIRT), accounting for both translational and rotational head movements [25, 26]. Resting-state functional MRI datasets were excluded if more than 20% of volumes exhibited FD greater than 0.5 mm.

### Infant-specific structural preprocessing and template normalization

3D T1-weighted images were processed using the Infant Brain Extraction and Analysis Toolkit toolbox (iBEAT), which has been specifically developed for anatomical segmentation of infant brain MRI during the first year of life [27]. Segmentation outputs were visually inspected for anatomical plausibility.

Individual T1-weighted images were non-linearly normalized to the age-matched UNC-BCP 4D infant brain atlas using the three-month T1-weighted template, providing a developmentally appropriate anatomical reference for infants aged 1 to 5.5 months [28] (https://www.nitrc.org/projects/uncbcp_4d_atlas/). Template normalization was performed using symmetric diffeomorphic normalization implemented in Advanced Normalization Tools (ANTs) [29]. Perfusion and resting-state functional MRI data were first independently registered to the corresponding native T1-weighted image using successive rigid and affine transformations. The resulting native-to-T1 transformations were then combined with the T1-to-template deformation fields to normalize each modality into three-month T1-weighted template.

### ASL data preprocessing

ASL images were reoriented to standard orientation using FSL (FMRIB Software Library) [30]. ASL image was co-registered to the corresponding native T1-weighted image using ANTs through successive rigid and affine transformations optimized with a Mutual Information similarity metric [31]. The resulting native ASL-to-T1 transformation was combined with the T1-to-template deformation fields obtained from the iBEAT-based structural segmentation and normalization procedure described above, and applied to transform ASL-derived CBF maps into the age-matched UNC-BCP three-month infant template space [28]. The accuracy of ASL-to-T1 registration was visually inspected to ensure appropriate alignment of cortical and subcortical anatomical landmarks.

CBF maps were automatically quantified in native space on the scanner console using a proton-density reference image included in the ASL sequence, in accordance with current ASL consensus recommendations [17]. The resulting CBF maps were normalized to each infant’s global mean and smoothed with a 6 mm Gaussian kernel prior to group-level comparisons using Statistical Parametric Mapping 12 (SPM12; https://www.fil.ion.ucl.ac.uk/spm). In this manuscript, CBF at rest refers to these global mean-normalized CBF maps.

### Preprocessing of resting-state functional MRI data

Resting-state functional MRI data underwent spatial preprocessing using FSL and ANTs. The first ten volumes were discarded to allow for magnetic field stabilization. Head motion correction was performed using MCFLIRT, followed by slice-timing correction.

A mean functional image was generated from the motion-corrected time series and co-registered to the corresponding native T1-weighted image using ANTs, following the same registration procedure as described for the ASL data. The native-to-T1 transformation was combined with the T1-to-template deformation fields obtained from the iBEAT-based structural segmentation and normalization procedure described above to normalize functional data into the age-matched UNC-BCP three-month infant template space [28]. Functional images were then smoothed using a 6 mm full width at half maximum Gaussian kernel implemented in Statistical Parametric Mapping 12.

### Definition of region of interest

A region-of-interest approach was used to investigate resting-state functional connectivity in brain regions showing significant group differences in CBF measured with ASL. Each cluster identified in the ASL analysis was defined as a region of interest. For each infant with PWS, the mean BOLD time series from each region of interest was extracted via the DPABI toolbox [32]. Pairwise correlations between region-of-interest time series were computed to assess functional connectivity between regions showing perfusion differences, and correlation coefficients were Fisher z-transformed before group-level analyses. In this manuscript, *functional connectivity* refers to these Fisher z-transformed correlations between pairs of regions showing significant perfusion differences in the ASL analysis.

### Outcome measures

The main exposure was a genetically confirmed diagnosis of PWS. Primary MRI outcomes included CBF at rest, measured with ASL and normalized to each infant’s global mean, and resting-state functional connectivity, estimated as Fisher z-transformed correlations between regions showing perfusion differences. The functional connectivity analyses were restricted to these regions and were considered exploratory. The secondary outcomes included oral-motor abilities assessed with the NOMAS, social functioning measured with the CIB, birth weight (z scores) and plasma ghrelin levels (acylated and unacylated, pg/mL) obtained from medical records. Associations with brain measures focused on CIB subscales reflecting child and dyadic functioning, as these dimensions best capture early affective and interactive development. Birth weight and ghrelin levels were included as clinically relevant measures available for all participants. VFSS data were not analyzed because reliable measurements could not be obtained for all infants, reflecting the challenges of assessing swallowing in early infancy.

### Statistical analyses

Differences in age and sex between groups were examined via independent-samples t tests and Fisher’s exact tests. Whole-brain voxelwise analyses of CBF at rest were performed in SPM12, which compared infants with PWS and controls within a gray matter mask (threshold 0.2). Age at MRI and sex were included as covariates in a multiple regression model. Statistical significance was set at *p* ≤ 0.05 with familywise error (FWE) correction at the voxel level, and significant clusters were reported with Montreal Neurological Institute (MNI) coordinates and cluster sizes.

Associations between imaging metrics (CBF at rest and functional connectivity) and clinical outcomes (NOMAS, CIB subscales, birth weight and plasma ghrelin level) were tested via linear regression models adjusted for age. For resting-state functional connectivity analyses, mean FD was additionally included as a covariate of non-interest to account for residual head motion effects. Associations between mean FD and age at MRI, as well as between mean FD and ROI-to-ROI functional connectivity coefficients, were also examined using linear regression. Model assumptions were verified for normality (Shapiro-Wilk test), linearity, homoscedasticity, and multicollinearity. To account for multiple comparisons, p-values from associations between clinical variables and ROI-to-ROI resting-state functional connectivity were corrected using the Benjamini-Hochberg false discovery rate (FDR) procedure across all ROI-to-ROI functional connectivity pairs. P-values from associations between clinical variables and regional CBF were corrected separately using the same procedure across all regional perfusion analyses. Only associations remaining significant after FDR correction are reported. Effect sizes were expressed as Cohen’s *f****²***, derived from the *R²* model, reflecting the proportion of variance explained by imaging and clinical measures. Statistical analyses were conducted in R (version 4.5.0).

## Results

### Study population

Of the 31 infants recruited (15 with PWS and 16 controls), four were excluded from ASL analyses (two with PWS, two controls), and three were excluded from resting-state functional MRI analyses (all with PWS) owing to poor image quality or preprocessing failure. Clinical measures were complete for NOMAS, CIB, birth weight and ghrelin levels, whereas data were missing for VFSS (*n* = 3).

After quality control, whole-brain perfusion analyses were performed on 13 infants with PWS (8 males; mean age = 2.7 ± 1.2 months) and 14 controls (8 males; mean age = 3.5 ± 1.1 months), corresponding to approximately one control per case, with no group differences in age (*p* = 0.14) or sex (*p* = 1.00) (see Table 1). Functional connectivity analyses were performed for 12 infants with PWS (7 males; mean age = 2.6 ± 1.09 months) (Fig. S1). Individual clinical characteristics for patients with PWS are summarized in Table 2.

**Table 1:** Demographic characteristics and head motion parameters in infants with PWS

Variable	Infants with PWS	Control group	*P*-value
ASL			
Age (months) mean ± SD; [range]	2.7 ± 1.05; [1.87–5.50]	3.4 ± 1.1; [1.5–5.50]	0.14
Sex (M ; F)	(8 ; 5)	(8 ; 6)	1.00
Resting-state functional MRI (PWS only)			
Age (months) mean ± SD; [range]	2.6 ± 1.09; [1.22–5.50]	-	-
Sex (M ; F)	(7 ; 5)	-	-
Mean FD (mm) mean ± SD; [range]	0.045 ± 0.005 [0.034–0.052]	-	-
Median FD mean ± SD; [range]	0.0412 ± 0.005 [0.032–0.048]	-	-
Volumes with FD > 0.3 mm (%). [range]	0.18 ± 0.50 [0.0–1.7]	-	-
Volumes with FD > 0.5 mm (%). [range]	0.00 ± 0.00 [0.0–0.0]	-	-
Participants excluded due to unusable rs-fMRI (n)	3	-	-

Age at MRI is reported as mean ± standard deviation with range. Sex is presented as number of males and females (M; F). Group differences in sex distribution were assessed using Fisher’s exact test. Head motion parameters during resting-state functional MRI are reported for the PWS group only, as resting-state functional MRI data were not acquired in control participants

*Abbreviations*: *ASL* arterial spin labeling, *FD* framewise displacement, *MRI* magnetic resonance imaging, *PWS* Prader-Willi syndrome

**Table 2:** Clinical characteristics of 15 infants with PWS

Patients					Neonatal examination			Oral motor and interactions skills									Biological data	
								Oral motor assessment			CIB subscore							
	Age	Sex	ASL	rs- fMRI	Birth weight (g)	Hypo- -tonia	Sucking disorder	Duration NGT	VFSS	NOMAS	Child social engagement	Child state	Dyadic negative	Dyadic reciprocity	Parental intrusiveness	Parental sensitivity	AG pg/mL	UAG pg/mL
1	5.5	F	+	+	1690	+	+	85	NA	8.0	3.375	4.5	2	3.545	1.727	4.235	120	903
2	1.87	M	+	+	1480	+	+	77	15	22.0	2.9	3.75	1.818	3.267	1.083	4.375	78	351
3	2.30	M	+	+	2060	+	+	82	12	9.0	1.625	3.75	2.444	2	2.182	3.235	178	947
4	1.22	F	-	-	3280	+	+	191	20	13.0	2.222	1.8	1.727	2.357	1.417	3.5	136	558
5	2.17	F	+	+	2060	+	+	34	NA	21.0	1.625	1.5	1.8	2.615	1.667	4.438	287	555
6	3.22	M	+	+	2850	+	+	80	19	11.0	2.875	3.25	1.556	3.833	1.231	4.684	309	290
7	2.76	F	+	+	1605	+	+	35	NA	19.0	1.75	1.75	2.333	2.727	1.75	4.389	177	382
8	1.91	M	+	+	1230	+	+	108	14	15.0	2.875	2	1.8	3.583	1.231	4.471	153	374
9	3.32	M	+	+	2420	+	+	34	18	8.5	1.9	3	1.833	2.5	1.462	4.556	41	534
10	1.97	F	+	+	2430	+	+	21	22	21.0	2.75	3	1.889	2.833	1.5	3.895	435	708
11	3.39	F	+	+	2990	+	+	63	11	14.0	1.818	2	1.75	2.333	1.909	3.588	122	187
12	1.54	M	+	-	2670	+	+	27	14	17.0	2.125	2.75	2.222	2.909	2.417	4.118	176	1380
13	2.23	M	+	+	2700	+	+	95	11	15.0	2.857	4.33	1.7	2.917	1.308	3.333	372	5
14	1.91	M	+	+	2760	+	+	0	12	8.0	3.375	4	2.222	2.818	2.167	2.563	218	557
15	0.93	F	-	-	2890	+	+	27	12	12.0	2	4	2.444	2	1.833	2.706	143	524

Variables are grouped into five domains: demographics, neonatal findings, oral motor/interaction skills, and biological data. Imaging availability (ASL, resting-state functional MRI) is indicated. Age is reported in months and birth weight in grams. Missing values are reported as “NA”

*Abbreviations:* *PWS* Prader-Willi syndrome, *MRI* magnetic resonance imaging, *ASL* arterial spin labeling; resting-state functional MRI, *NGT* nasogastric tube, *VFSS* videofluoroscopic swallowing study, *NOMAS* Neonatal Oral-Motor Assessment Scale, *CIB* Coding Interactive Behavior, *AG* acylated ghrelin, *UAG* unacylated ghrelin

Regarding head motion during resting-state functional MRI acquisition, the PWS cohort exhibited low in-scan motion (see Table 1 and Table S1), with a median mean FD of 0.045 (range: 0.034 to 0.051). The proportion of volumes exceeding an FD threshold of 0.3 mm was minimal (median = 0%, maximum = 1.7%), and no volumes exceeded an FD threshold of 0.5 mm.

Mean FD was not significantly associated with age at MRI (*p* = 0.49) or with any ROI-to-ROI functional connectivity coefficients (all FDR-corrected *p* ≥ 0.05).

**Fig. 1:**
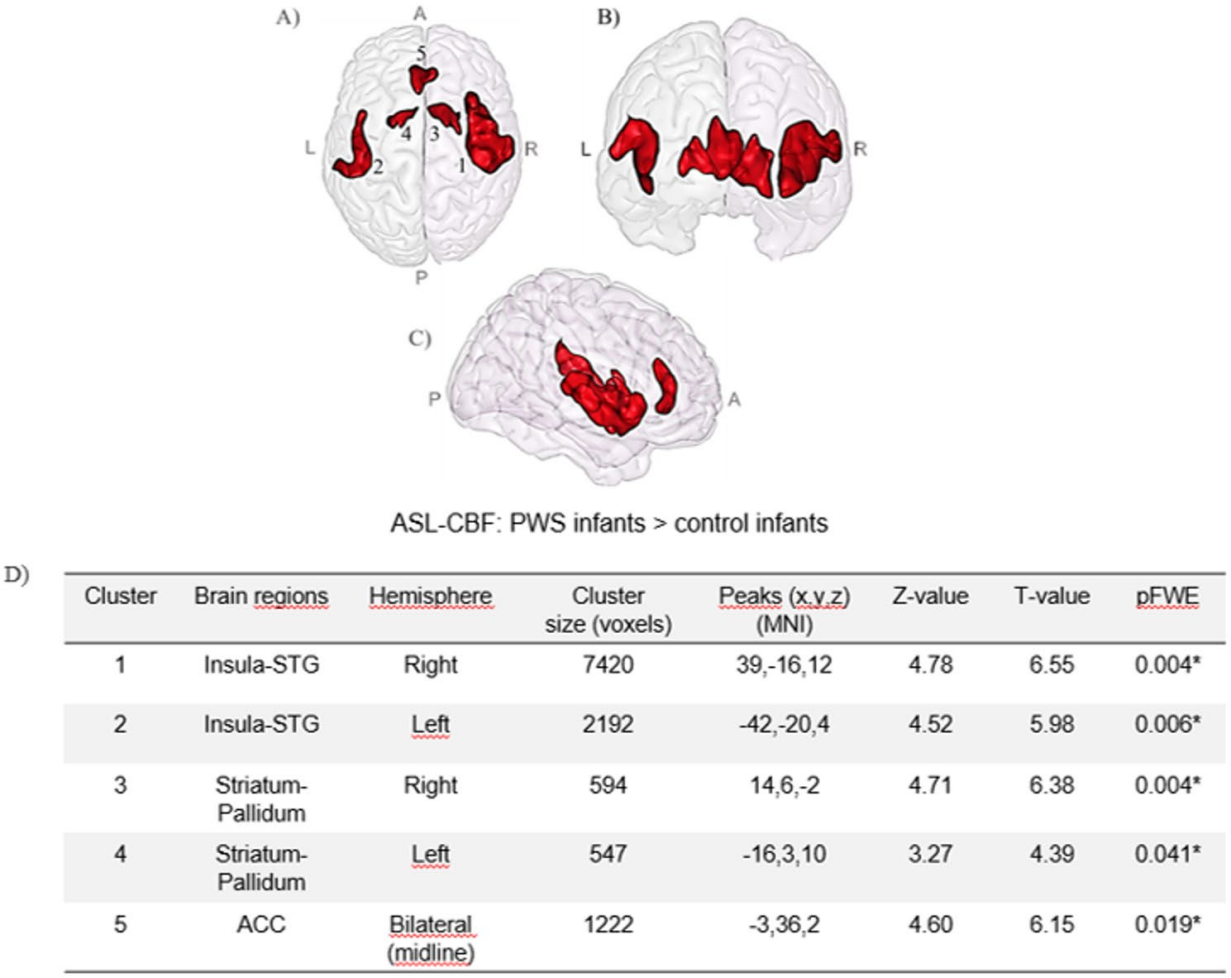
Brain regions showing significant hyperperfusion in infants with Prader-Willi syndrome (PWS) compared with controls. **A**-**C** Significant clusters (red) overlaid on axial (**A**), coronal (**B**), and sagittal (**C**) views of gray matter in the infant template space. **D** Peak coordinates and corresponding statistics. *Abbreviations: A*,* anterior; ACC*,* anterior cingulate cortex; L*,* left; P*,* posterior; PWS*,* Prader-Willi syndrome; R*,* right; STG*,* superior temporal gyrus*

Whole-brain voxelwise analyses revealed five clusters with significantly increased CBF in infants with PWS compared with controls (*p* ≤ 0.05, FWE-corrected), including the bilateral insula extending into the superior temporal gyrus (STG), the bilateral striatum-pallidum, and a midline cingulate cluster (Fig. 1). For clarity, these clusters are hereafter referred to as the insula-STG, ACC, and striatum-pallidum clusters. No regions presented a decreased CBF.

**Fig. 2:**
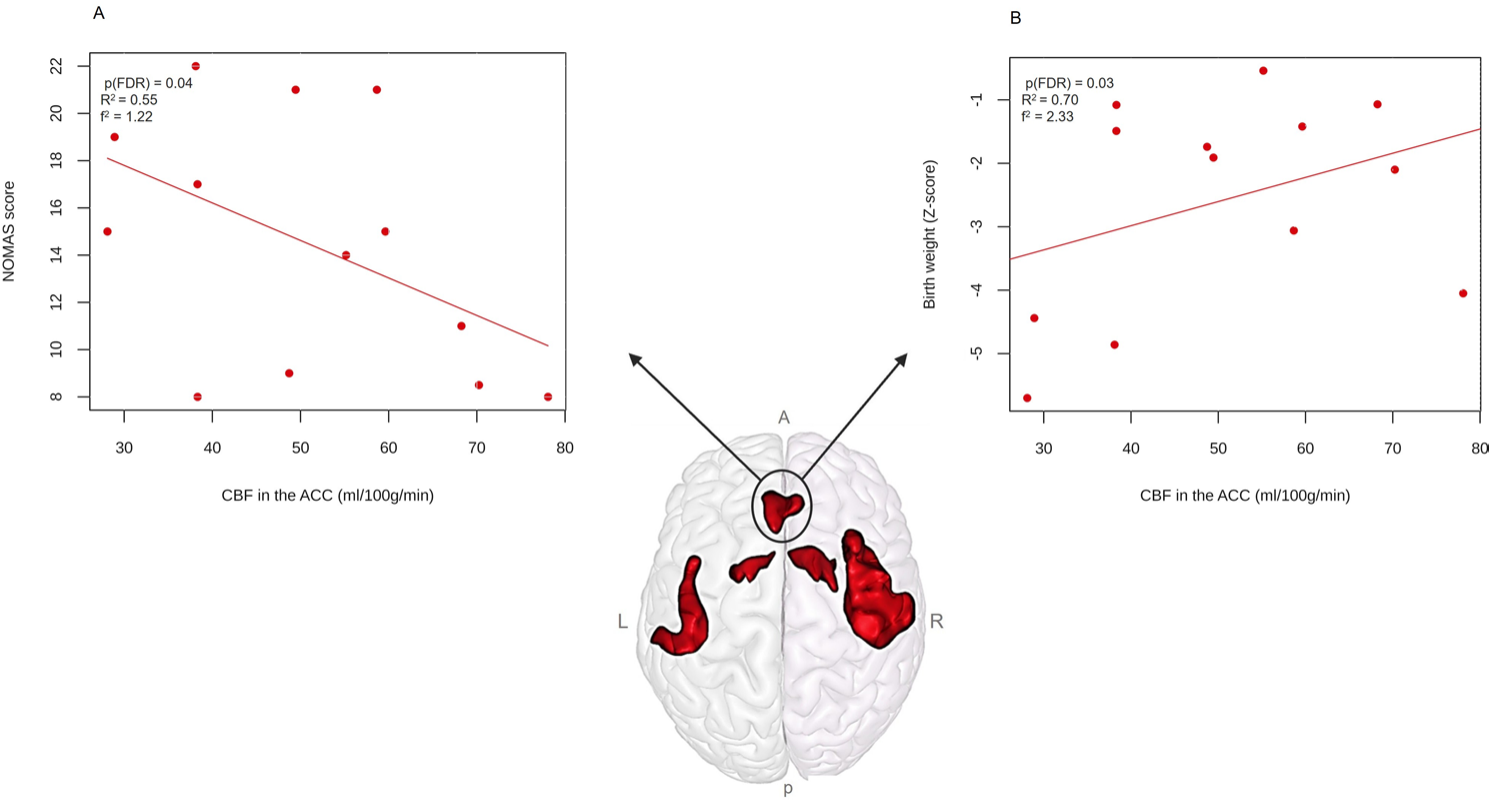
Associations between anterior cingulate cortex (ACC) perfusion and clinical measures in infants with PWS. (**A**) Association between CBF at rest in the ACC and oral-motor performance (Neonatal Oral-motor Assessment Scale (NOMAS) score; (**B**) Association between CBF at rest in the ACC and birth weight. The lower image shows the ACC cluster in the infant template space. P-values are Benjamini-Hochberg FDR-corrected for multiple comparisons across regional CBF-clinical associations. *Abbreviations: A*,* anterior; ACC*,* anterior cingulate cortex; L*,* left; NOMAS*,* Neonatal Oral-motor Assessment Scale; P*,* posterior; PWS*,* Prader-Willi syndrome; R*,* right*

**Fig. 3:**
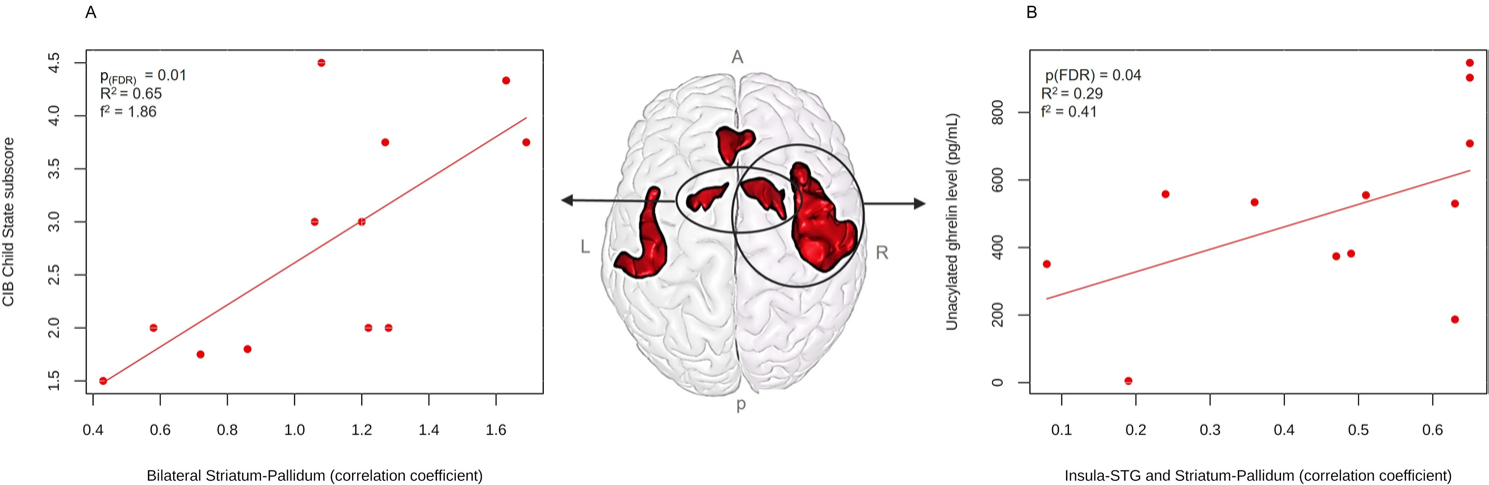
Associations between functional connectivity and clinical measures in infants with Prader-Willi syndrome (PWS). **A** Association between striatum-pallidum functional connectivity and the Child State subscale of the Coding Interactive Behavior (CIB). **B** Association between insula-superior temporal gyrus (STG) and striatum-pallidum functional connectivity and unacylated ghrelin levels. P-values are Benjamini-Hochberg FDR-corrected for multiple comparisons across all ROI-to-ROI resting-state functional connectivity pairs. *Abbreviations: A*,* anterior; CIB*,* coding interactive behavior; L*,* left; P*,* posterior; PWS*,* Prader-Willi syndrome; R*,* right; STG*,* superior temporal gyrus*

CBF at rest in the ACC was significantly negatively associated with NOMAS scores (*β* = -1.37, p_(FDR)_ *=* 0.04, *R²* = 0.55, f² = 1.22) and positively associated with birth weight (*β* = 4.34, p_(FDR)_ *=* 0.03, *R²* = 0.70, f² = 2.33) (Fig. 2). Within the PWS group, after adjustment for age at MRI and mean FD, resting-state functional connectivity between the left and right striatum-pallidum was significantly positively associated with the CIB Child State subscore (β = 1.98, p_(FDR)_ = 0.01, R² = 0.65, f² = 1.86). Unacylated ghrelin levels were also significantly positively associated with the Insula-STG and Striatum-Pallidum functional connectivity (β = 0.0004, p_(FDR)_ = 0.04, R² = 0.29, f² = 0.41) after adjustment for age at MRI and mean FD (Fig. 3). No other significant associations were found. Reported p-values for associations with resting-state functional connectivity and regional CBF correspond to values corrected for multiple comparisons using the Benjamini-Hochberg FDR procedure applied separately within each analysis.

## Discussion

This multimodal MRI study provides the earliest evidence of altered brain function in PWS during the first six months of life. Compared with controls, the PWS group presented marked increases in CBF at rest within the insula-STG, ACC, and striatum-pallidum. Within these circuits, ACC perfusion at rest is related to both oral-motor function and birth weight in these infants. Functional connectivity within perfusion-altered regions was further associated with social functioning and unacylated ghrelin, a circulating hormone related to metabolism.

Whole-brain analyses revealed early hyperperfusion within key regions involved in hunger regulation, motivational control, and reward processing, as previously reported in older individuals with PWS. This pattern, encompassing the insula-STG, ACC and striatum-pallidum, indicates early alterations in circuits supporting feeding, emotional, and social functions, which represent core features of the PWS phenotype [4]. Importantly, these perfusion differences were already detectable within the first months of life, highlighting early functional brain alterations emerging before the onset of clinical symptoms. The insula, ACC, and striatum form a core feeding network with complementary roles: the insula integrates hunger-satiety signals [21], the ACC regulates motivational control [22], and the striatum-pallidum drives food-seeking behavior [23]. Therefore, altered perfusion within these regions may reflect early imbalances in circuits regulating satiety, impulse control, and reward-driven feeding, potentially contributing to the later development of hyperphagia. In addition to feeding, the insula also contributes to pain processing [33], and early hyperperfusion in this region may be related to the atypical pain perception often described in PWS. The ACC, which is central to emotional regulation and conflict monitoring, could likewise be involved in the later emergence of the poor frustration tolerance and emotional dysregulation frequently observed in this population. Consistent with recent findings in other rare neurodevelopmental disorders, such as Sturge-Weber syndrome, where ASL has been used to identify early perfusion abnormalities, these results further support the potential of ASL as a sensitive marker of early brain dysfunction [34].

The observed hyperperfusion also extends to regions, such as the insula and STG, that are part of the social brain and play a central role in social perception and affective communication [35]. These anatomically adjacent and functionally connected areas jointly contribute to early social processing. In infants with PWS, increased perfusion in these social brain regions may be associated with the later emergence of social and communication difficulties. Interestingly, this pattern is consistent with findings in older individuals with PWS, in whom SPECT imaging revealed hypoperfusion in the ACC and STG-regions that showed increased perfusion in our infant cohort [36]. This pattern may reflect a developmental shift from early hyperperfusion to later hypoperfusion, highlighting the importance of interpreting functional brain changes within a developmental framework. Longitudinal imaging will be essential to determine whether these early perfusion alterations persist, normalize, or evolve with age and whether they predict later social and communication outcomes. Comparisons with idiopathic autism, where reduced perfusion in the superior temporal sulcus has been associated with social communication deficits [37], could further help identify which of these changes are specific to PWS and which reflect mechanisms shared across neurodevelopmental disorders.

To further understand the developmental relevance of these findings, we examined associations between regional brain perfusion and functional connectivity and early clinical measures in infants with PWS. Perfusion in the ACC at rest was associated with oral-motor function, which is consistent with its role in coordinating motor and attentional processes during early sucking [38], and with birth weight, suggesting a link between early metabolic regulation and later vulnerability to hyperphagia. Functional connectivity analyses based on associations with clinical measures, conducted only in infants with PWS, provided complementary information despite the absence of a control group. Interhemispheric functional connectivity between the striatum and pallidum was associated with the CIB Child State subscale, reflecting early affective and interactive functioning. This may indicate that temporal synchronization between the bilateral basal ganglia contributes to social regulation during early neurodevelopment. In addition, functional connectivity between the insula-STG and the striatum-pallidum was associated with unacylated ghrelin, a peptide involved in metabolism and neurodevelopment [39]. This association suggests an early link between metabolic signals and neural circuits involved in feeding and social behavior in individuals with PWS. Together, these exploratory findings highlight early functional patterns within basal and insular-temporal networks and emphasize the value of integrating neuroimaging with clinical and biological measures to advance the early understanding of PWS.

The limited sample size represents the main constraint of this study, reflecting the inherent difficulties of enrolling and scanning genetically confirmed infants with PWS under six months of age without sedation. Such sample sizes are consistent with early-stage neurodevelopmental MRI studies in very young infants, as stated in Hanspach et al. [19]. Despite this limitation, the observed effect sizes, together with stringent correction for multiple comparisons across all analyses and their alignment with previous findings, lend further support to the developmental relevance of our results. Additional limitations include the retrospective selection of controls, which was addressed by restricting inclusion to infants with normal scans and confirmed typical development at follow-up. Resting-state functional connectivity analyses were also limited to ASL-defined regions showing between-group perfusion differences and should therefore be interpreted as exploratory, even though they revealed clinically relevant associations. Moreover, interpreting associations between imaging and clinical variables at this early developmental stage remains challenging, as the clinical manifestations of PWS are not yet fully expressed in infancy. We therefore remain cautious in interpreting these associations, which may reflect early neural processes that precede, rather than result from, later-emerging symptoms. To ensure standardized assessment, a single experienced pediatric endocrinologist conducted all evaluations using validated scales appropriate for infants with PWS.

This study provides the earliest evidence of altered brain function in infants with PWS during the first months of life, before hallmark symptoms are fully clinically evident. Multimodal MRI revealed hyperperfusion in key regions, together with exploratory findings on functional connectivity associated with feeding, social functioning, and growth and metabolism (birth weight and ghrelin levels). These results suggest that perfusion-based measures could support developmental monitoring in PWS and emphasize the importance of longitudinal neuroimaging studies to establish their predictive significance for later outcomes.

## Supplementary Information

Additional file 1: Supplementary Figure S1. Flow diagram of participant inclusion and exclusion for ASL and resting state functional MRI analyses.

Additional file 2: Supplementary Table S1: Head motion parameters in infants with PWS. FD values are reported for each participant during resting-state functional MRI acquisition. Abbreviations: FD, framewise displacement; MRI, magnetic resonance imaging; PWS, Prader-Willi syndrome.

Additional file 3: Supplementary Table S2: Clinical characteristics of control infants included in the ASL analyses. Age at MRI is reported in months. Sex is presented as male (M) or female (F). Clinical indications for MRI are provided for each control participant. Abbreviations: MRI, magnetic resonance imaging; M, male; F, female.

Additional file 4: Supplementary Figure S2. Quality of image coregistration.

## Data Availability

The datasets used and/or analyzed during the current study are available from the corresponding author upon reasonable request.

## Abbreviations

ACC: Anterior cingulate cortex
ANTs: Advanced normalization tools
ASL: Arterial spin labeling
CBF: Cerebral blood flow
CIB: Coding interactive behavior
FD: Framewise displacement
FDR: False discovery rate
FWE: Family-wise error
iBEAT: Infant Brain Extraction and Analysis Toolkit
MCFLIRT: Motion Correction using FMRIB’s Linear Image Registration Tool
MNI: Montreal Neurological Institute
MRI: Magnetic resonance imaging
NOMAS: Neonatal oral-motor assessment scale
PWS: Prader-Willi syndrome
ROI: Region of interest
STG: Superior temporal gyrus
VFSS: Videofluoroscopic swallowing study
