# Supplementary material for: Early neurodevelopmental brain perfusion abnormalities and functional connectivity findings in infants with Prader-Willi syndrome
Source: J Neurodev Disord. 2026 Apr 6;18:28. doi: 10.1186/s11689-026-09690-4 (PMC13188529; doi:10.1186/s11689-026-09690-4)
Supplement: Supplementary file 1 — Additional file 1: Supplementary Figure S1. Flow diagram of participant inclusion and exclusion for ASL and resting state functional MRI analyses. [file 11689_2026_9690_MOESM1_ESM.pdf]

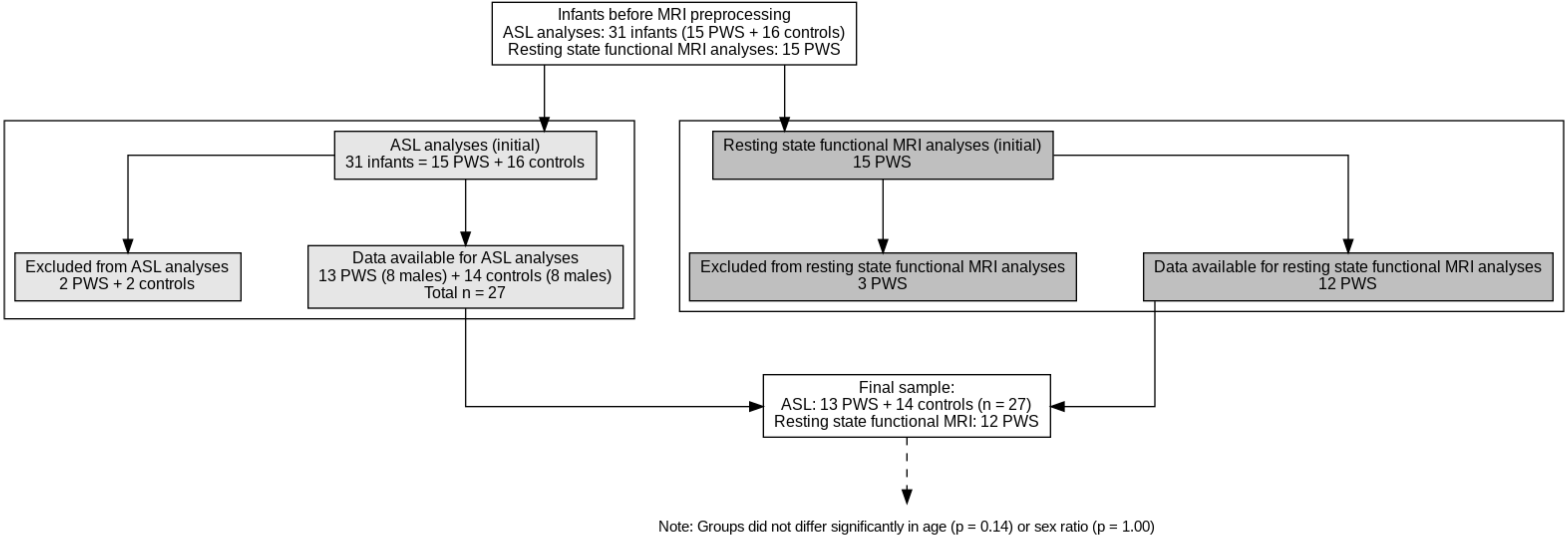
Infants before MRI preprocessing
ASL analyses: 31 infants (15 PWS + 16 controls)
Resting state functional MRI analyses: 15 PWS
ASL analyses (initial)
31 infants = 15 PWS + 16 controls
Excluded from ASL analyses
2 PWS + 2 controls
Data available for ASL analyses
13 PWS (8 males) + 14 controls (8 males)
Total n = 27
Resting state functional MRI analyses (initial)
15 PWS
Excluded from resting state functional MRI analyses
3 PWS
Data available for resting state functional MRI analyses
12 PWS
Final sample:
ASL: 13 PWS + 14 controls (n = 27)
Resting state functional MRI: 12 PWS
Note: Groups did not differ significantly in age (p = 0.14) or sex ratio (p = 1.00)
