# Supplementary material for: Early neurodevelopmental brain perfusion abnormalities and functional connectivity findings in infants with Prader-Willi syndrome
Source: J Neurodev Disord. 2026 Apr 6;18:28. doi: 10.1186/s11689-026-09690-4 (PMC13188529; doi:10.1186/s11689-026-09690-4)
Supplement: Supplementary file 2 — Additional file 2: Supplementary Table S1: Head motion parameters in infants with PWS. FD values are reported for each participant during resting-state functional MRI acquisition. Abbreviations: FD, framewise displacement; MRI, magnetic resonance imaging; PWS, Prader-Willi syndrome. [file 11689_2026_9690_MOESM2_ESM.docx]

| Infants with PWS | Mean FD  (mm) | Median FD  (mm) |
| --- | --- | --- |
| 1 | 0.0445 | 0.0424 |
| 2 | 0.0488 | 0.0429 |
| 3 | 0.0431 | 0.0347 |
| 4 | 0.0446 | 0.0409 |
| 5 | 0.0313 | 0.0452 |
| 6 | 0.0338 | 0.0319 |
| 7 | 0.0449 | 0.0424 |
| 8 | 0.0407 | 0.0341 |
| 9 | 0.0425 | 0.0384 |
| 10 | 0.0503 | 0.0477 |
| 11 | 0.0483 | 0.0453 |
| 12 | 0.0482 | 0.0468 |
