# Supplementary material for: Early neurodevelopmental brain perfusion abnormalities and functional connectivity findings in infants with Prader-Willi syndrome
Source: J Neurodev Disord. 2026 Apr 6;18:28. doi: 10.1186/s11689-026-09690-4 (PMC13188529; doi:10.1186/s11689-026-09690-4)
Supplement: Supplementary file 3 — Additional file 3: Supplementary Table S2: Clinical characteristics of control infants included in the ASL analyses. Age at MRI is reported in months. Sex is presented as male (M) or female (F). Clinical indications for MRI are provided for each control participant. Abbreviations: MRI, magnetic resonance imaging; M, male; F, female. [file 11689_2026_9690_MOESM3_ESM.docx]

| Control ID | Age at MRI | Sex | Clinical indication for MRI |
| --- | --- | --- | --- |
| 1 | 3.16 | M | Hemifacial capillary malformation without Sturge-Weber |
| 2 | 5.08 | M | Hemifacial capillary malformation without Sturge-Weber |
| 3 | 5.5 | F | Hemifacial capillary malformation without Sturge-Weber |
| 4 | 3.6 | F | Nystagmus |
| 5 | 3.2 | F | Nystagmus |
| 6 | 2.7 | F | Nystagmus |
| 7 | 4.15 | F | Suspected Horner syndrome |
| 8 | 5.01 | M | Exotropia |
| 9 | 2.16 | M | Strabismus |
| 10 | 2.04 | M | Strabismus |
| 11 | 1.5 | M | Strabismus |
| 12 | 2.53 | M | Strabismus |
| 13 | 4.05 | M | Congenital unilateral ptosis |
| 12 | 5.05 | F | Preoperative assessment of retroauricular mass |
