# Supplementary figures and images for: Early neurodevelopmental brain perfusion abnormalities and functional connectivity findings in infants with Prader-Willi syndrome
Source: J Neurodev Disord. 2026 Apr 6;18:28. doi: 10.1186/s11689-026-09690-4 (PMC13188529; doi:10.1186/s11689-026-09690-4)

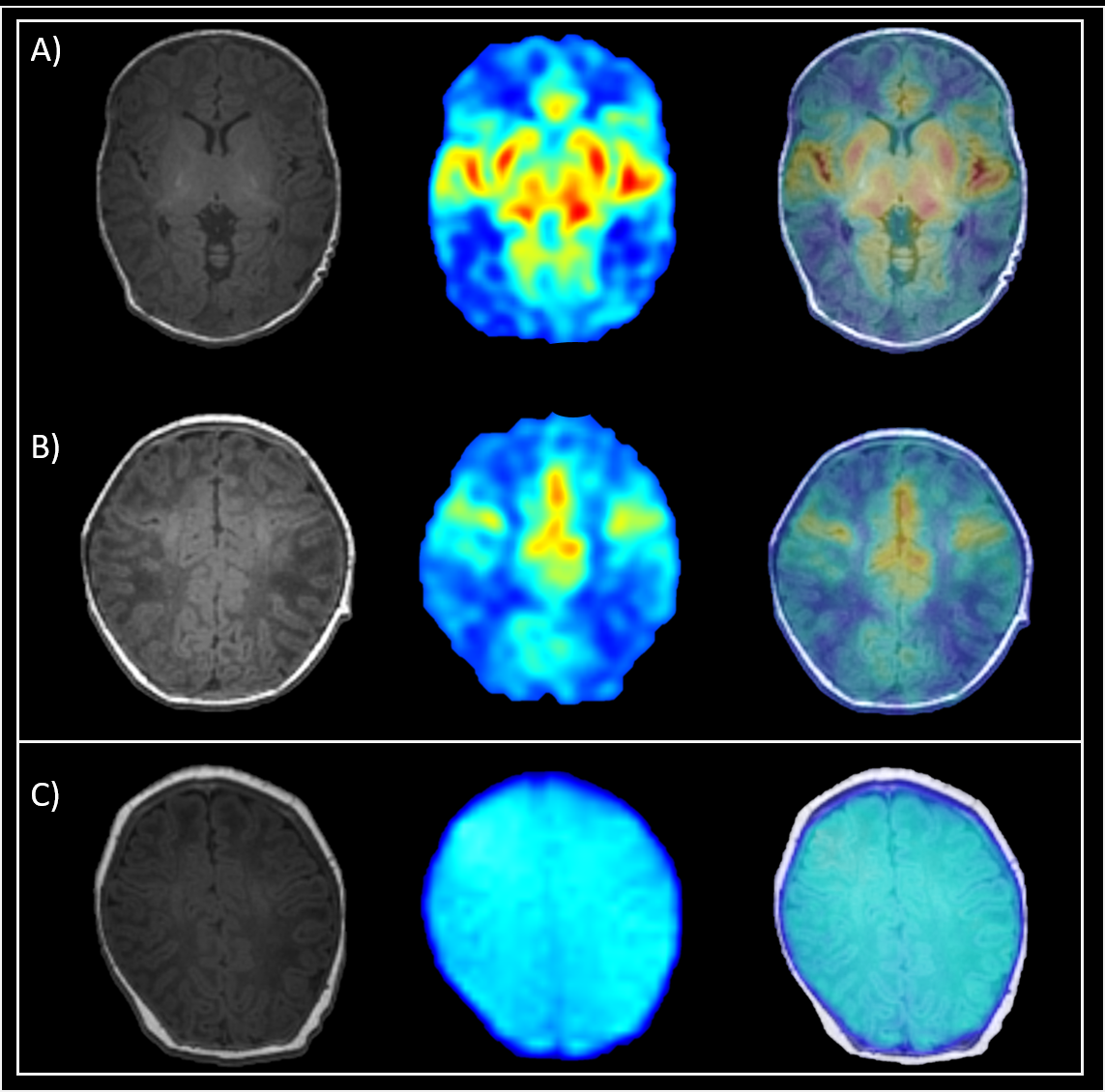

Supplement: Supplementary file 4 — Additional file 4: Supplementary Figure S2. Quality of image coregistration. [file 11689_2026_9690_MOESM4_ESM.tiff]
